# Methodological approaches on synergies and trade-offs within the 2030 Agenda

**DOI:** 10.1016/j.isci.2024.111100

**Published:** 2024-10-05

**Authors:** Aliya Assubayeva, Jorge Marco

**Affiliations:** 1 Centre for International Development and Environmental Research, Justus Liebig University Giessen, 35390 Giessen, Germany; 2 Department of Economics, University of Girona, 17003 Girona, Spain; 3 Department of Economics, University of Los Andes, Bogotá 11171, Colombia

**Keywords:** Environmental science, Environmental policy

## Abstract

The world is lagging in achieving the 2030 Agenda’s 17 Sustainable Development Goals (SDGs), emphasizing social, economic, and environmental dimensions. Meeting one target may either help or hinder meeting another, but the high number of potential interactions complicates the evaluation of synergies and trade-offs. Here, we focus on the water-energy-food nexus to assess how the knowledge of SDG interlinkages has been operationalized to inform policymaking. Specifically, we review the effectiveness of research methodologies, such as correlation analysis, network analysis, meta-analysis and literature reviews, expert-based assessments, and integrated assessment models in characterizing SDG interlinkages. Most studies indicate that synergies are more prevalent than trade-offs, but they have seldom analyzed whether such infrequent trade-offs might nevertheless have a greater impact on sustainable development. Further, existing methods do not always reveal the directionality and strength of SDG interactions or consider projections of future interlinkages such as transboundary and intergenerational spillovers. In this context, it may be worth revisiting earlier definitions of sustainable development that prioritized intergenerational aspects and future needs.

## Graphical abstract

**Figure:**
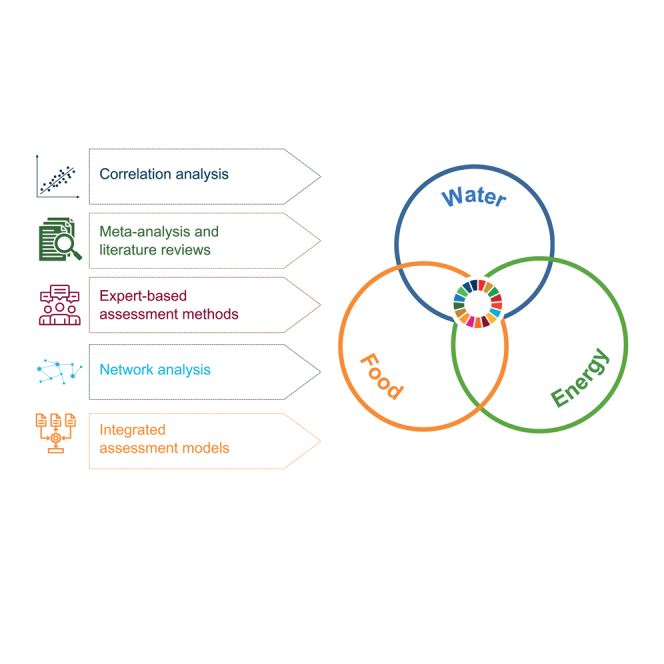

## Introduction

The 2030 Agenda emphasizes the equal importance and indivisibility of all Sustainable Development Goals (SDGs), which address sustainable development’s social, economic, and environmental dimensions. The 17 SDGs comprise 169 targets and 232 indicators to achieve the 2030 Agenda. However, progress in some SDGs, such as those related to economic development, may hinder environmental SDGs. Conversely, improvements in some SDGs may positively affect other SDGs. The saliency of the 2030 Agenda on conflicting goals is observed not only in rhetoric but also in implementation. Certain ministries/agencies may only focus on achieving SDGs related to their area of responsibility, neglecting the potential impacts on the progress of other SDGs. To avoid siloed thinking, it is recommended to identify and quantify SDG interlinkages, especially due to limited capacities, resources, and technologies to simultaneously improve and invest in the progress of all SDGs and targets.^1^^,^^2^^,^^3^^,^^4^^,^^5^^,^^6^ The world is not on track to meet all SDGs, and only 16% of the SDG targets can be achieved globally by 2030.^7^^,^^8^ Analyzing interactions between SDGs can help minimize trade-offs and negative externalities while identifying synergies and positive spillover effects can leverage the 2030 Agenda and climate actions.^3^^,^^4^^,^^9^^,^^10^^,^^11^ Accounting for synergies and acknowledging trade-offs holds the potential to strengthen policy integration and coherence by contextualizing target interactions, providing integrated assessments of SDGs, and supporting policy prioritization and governance in short, medium, and longer planning.^6^^,^^9^^,^^12^

Academic publications on SDG interlinkages have increased significantly since 2015.^9^^,^^13^ Yet, the current literature on SDG interactions is mainly about the conceptualization of SDG interlinkages,^9^^,^^14^ aiming to explore and identify these interactions that are classified into reinforcing SDG interactions, synergies or positive interactions, and constraining SDG interactions, trade-offs or negative interactions.^5^ Other classifications are based on the goals and targets being either synergistic and reinforcing, symbiotic and dependent on each other, or antagonistic and obstructing each other; reinforcing, conflicting/constraining, or mixed^15^; and unidirectional, bidirectional, or asymmetrical.^16^ When assessing interactions between SDGs, one important question is how progress in one goal influences progress in another. The level of analysis varies from the SDG goal to the SDG targets and the SDG indicators. Data sources primarily consist of secondary data from official databases and scientific literature and primary data from surveys, interviews, and focus group discussions.^8^^,^^17^^,^^18^^,^^19^

Operationalization of SDG interlinkages, whether quantitative or qualitative analytical frameworks, to account for synergies and trade-offs among SDGs or targets within the 2030 Agenda remains challenging due to the dimensionality problem. There are 272 potential interactions among SDGs and 28,392 potential interactions among targets. In response to the challenge of high dimensionality, scholars have focused on analyzing target interactions within specific subsets of SDGs. This approach, known as the “nexus approach”,^20^ specializes in assessing mutually limiting (trade-off) and mutually reinforcing (synergy) interactions that may arise from initiatives toward achieving prioritized goals within the 2030 Agenda. Despite the direct and indirect trade-offs between economic and environmental SDGs or trade-offs in climate action and socio-economic SDGs,^10^^,^^21^ the water-energy-food (WEF) nexus has received considerable attention in the literature on nexus approaches, as an annual publication growth rate of 24.57% between 2015 and 2021,^22^ due to increasing resources scarcity, competing and conflicting interests for resources, and preventing negative consequences of their use.^6^^,^^23^^,^^24^ The WEF nexus involves three SDGs and 21 targets, leading to 420 potential interactions among these targets. The resource nexus refers to the critical linkages between two or more natural resources used in delivery chains toward provision systems for water, energy, food, land, and context-specific resource availability.^25^

The “indivisible whole” attribute of the 2030 Agenda assumes that goals and targets mutually support sustainable development; however, there are trade-offs and constraints among SDGs and targets. Since there are already several studies reviewing scholarly literature on SDG interactions,^9^^,^^13^^,^^16^^,^^26^^,^^27^ this paper focuses on exploring the operationalization of SDG interlinkages by critically reviewing quantitative and qualitative methodologies applied in assessing SDG interlinkages, including longitudinal and cross-sectional analysis, network analysis, meta-analysis, expert-based assessments, and integrated assessment models (IAMs). The following sections discuss the advantages and limitations of commonly used analytical frameworks on SDG interlinkages, with examples of the WEF nexus. We identify the major challenges associated with these methods to improve the accuracy and reliability of SDG interlinkage analyses. These challenges stem from data gaps, varying data sources, non-linear and interdependent relationships, context-specific factors, and temporal dependence and dynamics. These challenges constrain the comparability and interpretability of findings on a global scale. The Discussion section provides recommendations for future research exploring SDG interlinkages across various contexts, domains, and analytical levels. This paper aims to provide valuable insights for those interested in producing policy-relevant results by examining and quantifying SDG interlinkages while addressing the limitations of each method and exploring the benefits of employing multiple methods. Future studies may benefit from employing a combination of methods depending on resource availability, research objectives, disciplinary perspectives, and the level of analysis. In addition to a critical review of research methods, this paper guides accessing global databases in the Appendix. The paper contributes to methodological advancements in the ongoing discourse on SDG interlinkages, raising awareness of the pros and cons of different methods and supporting policy-making efforts to strengthen SDG synergies and transform trade-offs into opportunities for sustainable development at local, national, and global scales.

## Critical review of research methods

### Correlation analysis

Quantitative methods have predominantly employed correlation analyses, whether cross-sectional^3^ or longitudinal,^5^ to characterize the interactions among SDG/target pairs as synergies (positive correlations), trade-offs (negative correlations), or neutral (non-significant correlation). Correlation analyses offer the advantage of simplicity in computation and interpretation. The nature of the interaction is determined by a best-fit regression line through the data points together with the coefficient of determination (R^2^) parameter. Cross-sectional analyses rely on the Pearson coefficient to assess interactions at a specific point in time, while longitudinal analyses utilize Spearman’s coefficient to assess how interactions develop over time. Typically, the correlation coefficient value of ±0.5 serves as the threshold for classifying the interaction as synergy (>0.5) or trade-off (<-0.5) among the SDG/target pair. An interaction is classified as neutral when the correlation coefficient is below this threshold.^3^^,^^28^

Correlation analyses have benefited from considerable advances in the availability of internationally standardized data. The number of indicators in the global SDG database increased from 115 in 2016 to 225 in 2023. Moreover, the proportion of conceptually clear indicators with good country coverage has increased significantly from 36% in 2016 to 66% in 2022.^29^ The increased availability of data facilitates a shift in the level of analysis from interactions among SDGs to a more focused examination of interactions among targets.^30^ The minimum unit of analysis is the country, and the scope of research studies is the comparison between countries or regions.^3^^,^^22^ However, it is worth noting that to address this information gap between available (66%) and desired (100%) indicators, the SDG Index (the SDG Index provides an annual assessment of SDGs progress in all 193 UN member states. It builds on a composite, peer-reviewed, statistically audited, and transparent methodology^8^), in its different regions, has used a combination of SDG indicators (referred to as “official indicators”) with other indicators from internationally recognized information sources.^31^ The latter, which we will refer to as “alternative indicators,” are not intended to replace the unavailable official indicators but rather to make visible information within the SDGs that would not have been possible to show otherwise. Divergent use of alternative indicators across regions introduces variability, complicating cross-regional, and longitudinal result comparisons. For example, Target 10.1 (Reduce Income Inequalities) does not specify which indicator should track progress in reducing income or wealth inequality.^32^ The Gini index and the Palma ratio are the two main indicators used by countries for this purpose. However, they provide different information and are not easily comparable.^33^ Regions focusing on extreme poverty or wealth concentration, like Latin America and Africa, tend to prefer the Palma ratio. Conversely, regions prioritizing broad-based economic growth and overall inequality reduction favor the Gini index for its comprehensive approach. To enhance comparability, a well-balanced analysis of inequality should consider multiple measures, such as using both indicators to monitor Target 10.1.^34^ Mair et al.^35^ extensively discuss the adoption of alternative indicators and their implications for monitoring sustainable development progress. Due to persisting data gaps and the absence of a unified methodology for assessing information coverage with official and alternative indicators, we remark that the most robust results will likely emerge from comparisons among countries within the same region. For instance, Hao et al.^36^ exemplify this approach in their comparative study of WEF security evaluations across five Central Asian countries.

Correlation analyses have two important limitations when assessing the significance of SDGs/targets based on their specific contributions to the advancement of sustainable development. First, they assume reciprocal influence, that is, the nature of interaction among (SDG/target) i and j, and j and i coincides, which may not always hold.^37^ In a recent study, Sušnik^19^ demonstrates that within the WEF nexus, analyzing causality between correlated pairs allows discerning the directionality and strength of influence, helping identify instances where one direction of influence is more pronounced. Despite its importance, the analysis of causality among correlated interactions has received little attention in the existing literature. Second, they represent the 2030 Agenda as a sequence of pairwise SDG/target interactions and do not consider the extent to which the structure (network) of these interactions, through spillover effects, influences progress toward sustainable development. In this context, spillover effects refer to the indirect influence resulting from interactions, i.e., the influence of i on k through the intermediary influence of j. Assessing the spillover effects of progress in a specific SDG/target within the intricate network of synergies and trade-off interactions can facilitate the integration of that SDG/target’s progress with overall sustainable development. Spillover effects challenge the assumption that every SDG/target holds identical theoretical potential to contribute to sustainable development.^3^^,^^38^^,^^39^

In the coming years, a major challenge lies in tackling the issue of reducing the heterogeneous distribution of data availability and quality across international databases, along with the variability that affects the comparability and interpretability of findings on a global scale. In particular, developing countries marked by limited infrastructure for data collection and management face the challenge of rapidly adapting and developing to meet the evolving needs of monitoring sustainable development performance. The Statistical Performance Indicators and Index (SPI) is the World Bank’s new official tool to measure a country’s statistical capacity.^40^ According to our observation, Latin American countries with national statistical systems adhering to best practices, as measured by the SPI, generally exhibit high data coverage in the SDG Index. In Appendix B, we list databases for SDG data and monitoring and some tools and packages for network and quantitative analysis discussed earlier.

Data gaps hinder comparisons among both SDGs/targets and countries. For instance, in Kroll et al.,^3^ SDGs 10 (Reduced Inequalities) and 12 (Responsible Consumption and Production) are excluded from correlation analyses due to insufficient data availability. Nilashi et al.^18^ advocate leveraging big data to enrich SDG datasets, address missing values in SDG indicators, and improve data quality and accuracy. While data enrichment through big-data analysis improves shareability and comparability, its adoption necessitates substantial financial resources for developing tools and data infrastructure. To address measurement gaps hindering local sustainable development monitoring, the UN Statistics Division and the United Nations Institute for training and research have introduced “StaTact,” an innovative tool to enhance accessibility to data, data usage, and data literacy. Once data are collected, the effectiveness of statistical methods relies on assumptions such as linearity and independence. Deviations from these assumptions can introduce biases into the analysis, potentially compromising the interpretability of findings. Therefore, the complexity of interlinkages among SDGs and spillover effects poses significant challenges due to nonlinear and multivariable SDG interactions, making causality elusive amid confounding variables and contextual intricacies. Moreover, the limited scope of analysis, primarily concentrated on countries and a select subset of SDG indicators, may compromise the generalizability of findings. This underscores the need for more comprehensive approaches beyond correlation analysis to enrich our understanding of pathways to sustainable development on a global scale.

### Network analysis

Network analysis maps direct and indirect interactions among SDGs or targets, examining their structure and the complex, often non-linear effects of their synergies and trade-offs. This method excels at identifying which SDGs or targets are most influential for overall progress and which may require additional attention to mitigate potential negative effects on sustainable development. Network analysis enhances policy prioritization and governance by accounting for direct and spillover effects, offering valuable insights for more effective decision-making. Although all SDGs and targets are important, their levels of influence may vary according to network effects. Policymakers should prioritize based on the relative influence of each SDG or target on overall progress.^3^^,^^41^ Representing the 2030 Agenda as a network currently faces two significant methodological limitations.

On the one hand, there is no definitive methodology for systematically ranking SDGs or targets (nodes) according to their network effects. In this context, “ranking” refers to ordering nodes based on their relative influence on overall progress within the network. Network-based rankings use centrality metrics such as degree centrality (influence based on the total number of direct connections), eigenvector centrality (influence based on the total influence of its direct and indirect connections), betweenness centrality (influence based on the capacity to act as a bridge between other nodes), and closeness centrality (influenced based on the speed of reaching all other nodes).^42^^,^^43^^,^^44^ Among these network metrics, eigenvector centrality (EC) stands out as the most recommended metric for determining the centrality or relative influence of a node.^45^ The EC metric considers not only the direct interactions to and from each node but also the interactions of interacting nodes (neighbors), as well as the neighbors of neighbors and beyond. Consequently, EC emerges as a comprehensive metric, capturing the relative influence of nodes throughout the entire network structure.^46^ However, the EC metric experiences the following two limitations within the context of the 2030 Agenda, where both synergistic and trade-off interactions among nodes exist.^47^ First, in undirected networks characterized by reciprocal interactions, the presence of trade-offs may lead to situations with non-dominant or repeated eigenvalues, resulting in the emergence of multiple linearly independent eigenvectors. In such cases, the centrality of nodes is arbitrarily assigned, leading to less robust results. Second, a single measure of centrality is insufficient in directed networks characterized by non-reciprocal interactions. Instead, two measures are necessary: one to identify nodes that produce the greatest positive/negative effects (referred to as “affecting”) and another to identify nodes that receive the greatest positive/negative effects (referred to as “affected”) within the network. Again, the EC metric suffers from robustness issues in its results. To address this methodological challenge, the use of the positive-negative centrality metric is recommended,^47^ which has demonstrated effectiveness in networks including both positive (synergies) and negative (trade-offs) interactions. Despite its potential, the positive-negative metric has received minimal attention in existing literature. To organize the results from network analysis effectively, priority should be given to policies that address the mitigation of unintended negative impacts in the pursuit of sustainable development and, for example, water/energy/food security evaluations within the WEF nexus. As a result, the nodes should be arranged in descending order based on the centrality scores, with a focus on the negative effects they either produce (affecting) or receive (affected). These scores serve as key rankings: a lower score, closer to zero, indicates more pronounced trade-offs produced or received by a specific node. Conversely, a higher centrality score exceeding one signifies greater synergies produced or received. A centrality score of one should be interpreted as the baseline for calculations. The total contribution of nodes to sustainable development can be quantified by combining the affecting and affected dimensions.^38^

On the other hand, network analysis condenses each node interaction into a singular value, making it effective when robust quantitative data on SDG interactions—such as statistical correlations, co-occurrence, or documented relationships—are available. This value indicates the strength of the interaction. However, qualitative analysis based on literature reviews or expert assessments often provides multiple values for node interactions, reflecting variations across countries, criteria, or over time.^37^^,^^48^^,^^49^ Network analysis is highly sensitive to the quantity and strength of synergies and trade-offs in evaluating node centrality. As a result, a critical challenge in network analysis is to identify an effective aggregation/comparative method capable of handling the multiple values associated with node interactions to improve the robustness of results. The emphasis in research currently lies in analyzing the sensitivity of results,^45^ with limited attention given to exploring methods for minimizing it. In this context, Ospina-Forero et al.^50^ suggest disregarding quantitative methods with theoretical assumptions less aligned with the nature of SDG data, along with those methods prone to generating relatively higher rates of falsely estimated SDG interactions. As previously mentioned, the high sensitivity to interaction values may result in the arbitrary assignment of node centralities and, therefore, policy prioritization and coherence. In addition to quantitative methods, expert-based assessments such as fuzzy cognitive maps (discussed in the next section) are valuable for understanding causal relationships, feedback loops, and uncertainties in node interactions over time. Integrating these qualitative insights with quantitative network analysis enables the simulation and testing of different policies and their structural impacts on SDGs and targets, facilitating the exploration of “what if” scenarios.^9^^,^^51^^,^^52^ The Analytic Network Process is gaining recognition as a multi-method approach that combines expert assessments with empirical data in a cross-impact matrix. This method not only facilitates the derivation of node rankings but also supports comprehensive policy evaluation based on structured data.^14^ Lastly, it is noteworthy that the temporal dependence of SDG interactions within a network may also be influenced by any existing policy intervention in place. Agent-based models and system dynamics models, such as the Integrated Sustainable Development Goals (iSDG) model, are well-suited for analyzing the complex (co)evolution of SDG networks and policy interventions.^53^ These two methods help create integrated frameworks, or “vertical mechanisms,” that predict how different levels of policies and SDG networks interact and influence each other, thereby designing effective pathways to ensure sustainable development and reduce time delays in achieving the 2030 Agenda.

### Expert-based assessment methods

Due to variability in data availability, quality of data, and context-specific SDG interlinkages, researchers often rely on qualitative methods such as questionnaires, interviews, and focus group discussions to gather expert opinions, stakeholder knowledge, and perspectives on contextual SDG interactions.^37^^,^^52^^,^^54^ Studies frequently apply Nilsson’s scoring system^55^ (see Appendix A) to organize, evaluate, and analyze the data. Nilsson et al.^55^ and Griggs et al.^37^ proposed a seven-point scale to assess SDG interactions. The scale is classified into positive (+1, +2, +3 for enabling, reinforcing, indivisible), consistent (0), and negative (−1, −2, −3 for constraining, counteracting, and canceling). Using Nilsson’s scoring through focus group discussions or online surveys, participants evaluate how progress in one SDG/target will influence another SDG/target. The collected data are then analyzed using descriptive statistics, cross-impact matrices, network analysis, and clustering analysis.^14^^,^^54^

To operationalize Nilsson’s scoring, the SDG Synergy Tool, an interactive platform in English and Spanish, was developed by the Stockholm Environmental Institute. Administrators can select SDGs or targets to conduct surveys to assess SDG interactions in a specific context and analyze data simultaneously using various visualization tools such as the cross-impact matrix, network analysis, and scoring with comments if mentioned by participants. This tool was applied in the case of SDG interactions in Sweden,^54^ Sri Lanka,^56^ Colombia,^57^ Mongolia, and in comparing these cases.^58^ It is particularly effective in raising awareness and promoting systems thinking, learning, and capacity building. However, a limitation of this method is that it can be time-consuming and become tedious and repetitive for participants after assessing several SDG interactions. Additionally, there may be varying understandings of Nilsson’s scoring even after introducing it before the survey. Even when focusing on a specific country and topic, participants noted differences in SDG interactions among regions, even within one country. Therefore, assigning a score of +2 or +1 would have different meanings for each expert/evaluator, making it difficult to generalize and determine average grades for certain SDG interlinkages. Weitz et al.^54^ recognized that the learning process and policy dialogue are more important in this method than the results/scoring themselves. The scales used cannot measure the strength of interactions, and it was observed that most interlinkages were positive or synergetic.

Multi-criteria decision-making methods aid in quantifying SDG interactions and support policymakers in setting priorities and providing policy advice.^14^^,^^59^ For example, Analytic Hierarchy Process or Analytic Network Process techniques are applied using online software for setting priorities and pairwise comparisons.^14^ The Delphi method is also applied to collect experts’ opinions and reach an agreement among panel members on areas with limited information and high uncertainties, such as water security.^60^ Additionally, fuzzy cognitive maps are applied to specify causal-effect links of the interdependent SDGs using Mental Modeler software based on the experts’ opinions.^51^ An interesting study compared SDG interconnections between experts using fuzzy cognitive maps with Monte Carlo simulations and results of IAMs.^52^ The results reveal that experts’ input shows more interconnections among SDGs than IAMs results; however, IAMs outputs set priorities among SDGs (ibid).

While many studies concentrate on specific SDGs or areas, such as the water-energy or water-climate nexus, some studies fail to explain the rationale behind selecting SDGs and targets. The analysis often lacks information on the temporal and spatial scale and whether experts conducted the assessments considering these scales. Additionally, expert judgments can vary due to differences in expertise, perceptions, and semantic meaning of the scores^14^ and the lack of information regarding the experts’ backgrounds, such as their expertise or experience, and who considered as experts in a specific study, limits the reliability of these assessments. Other barriers include small sample sizes, limited stakeholder participation, and the time-consuming process. Future studies should minimize any biases in selecting SDGs and experts. Bringing together different stakeholders and experts can help create common ground and understanding of the impact of certain policies on other areas/SDGs, and this method can be useful for preliminary and general assessments. Co-creation of scenarios with non-academic actors,^52^ communication, verification, and validation of findings, for example, from quantitative methods via focus groups and workshops with experts, are key for policy and practical relevance of these SDG assessments.

### Meta-analysis and literature reviews

Due to the growing number of research publications about SDG interactions since 2015, literature reviews and document analysis are widely used research methodologies for exploring SDG interlinkages. Studies employ different approaches, such as systematic reviews, bibliometric reviews, and meta-analyses of peer-reviewed publications and gray literature. They focus on certain thematic areas and a combination of several SDGs. Water, energy, and land are often used as leverage points—resources that connect several SDGs and provide a system understanding of interlinkages. Examining the linkages between water and the SDGs is more holistic than analyzing only SDG 6 (Clean Water and Sanitation), argue Taka et al.^27^ For example, water and SDGs topics include water quality,^61^^,^^62^ water security,^27^ water-associated ecosystems,^63^ health,^64^ groundwater,^15^ and water pollution.^65^ The geographical scale of analysis varies, ranging from country-level studies such as those conducted in South Africa,^63^ Australia,^64^ and China,^65^ to river basin scale studies^66^ and global studies. It is important to note that the interaction between SDGs is context-specific and depends on the availability of resources, technology, and state capacity; yet, many studies fail to mention temporal and spatial scales of analysis, resulting in a vague and broad analysis.

Some literature reviews analyze interactions thematically or descriptively, while others use scoring approaches such as Nilsson’s scoring typology (described in the previous section). Literature reviews are often combined with expert surveys and authors’ scoring. Some studies examining the interactions between SDGs in broad concepts, such as water resilience, sustainable agriculture, biodiversity conservation, ecosystem services, and many more, fail to define these concepts. This lack of conceptual clarity can lead to varying assessments among studies and scholars depending on their perspectives and disciplines. The discussion remains focused on descriptive interactions, synergies, and trade-offs and lacks directionality, specifically whether the dependencies are unidirectional, bidirectional, or asymmetrical.^16^

Researchers can benefit from this method due to the availability of growing publications on SDG interlinkages. The literature search is primarily conducted in English. However, using bibliometric databases such as Scopus, Science Direct, Google Scholar, and Web of Science or lack of access to them may introduce bias in literature selection. The methodology section briefly mentions keyword searches and databases without sufficient explanation or justification for selecting certain SDGs and keywords. Machine learning techniques and software are available to automate text analysis and auto-coding. Still, there are some caveats: the variability of textual data are compounded by semantic understanding issues arising from the need to integrate diverse knowledge into these models to enhance interpretability.

Appendix A summarizes the criteria of quantitative and qualitative methods in a seven-point scale for assessing interactions between pairs of SDGs/targets, adapted from Nilsson et al.^55^ It provides an interpretation of interaction strengths and how different methods attempt to classify based on criteria in regression analysis, correlation analysis, comparative analysis, literature review, and expert judgments.

### Integrated assessment models

IAMs are important for quantifying the relationships between human activities, economic development, and environmental impacts over time. They are particularly well-suited for assessing climate scenarios and technological developments, as well as for evaluating synergies and trade-offs among SDGs to enhance policymaking, assess pathways to societal transitions (e.g., lifestyle changes or decentralized governance), and support informed decision-making.^11^^,^^21^^,^^24^ IAMs have been used extensively to analyze linkages within nexus approaches.^10^ IAMs integrate multidisciplinary knowledge and large databases and vary in scale (national, regional, global) and sector-specific models, requiring extensive expertise and resources.

For example, the International Futures model was applied to analyze three policy pathways (global technology, decentralized governance, and consumption change) in achieving selected human development SDG targets by 2030 and 2050.^24^ The IAM framework of integrating the multiregional energy-economy-land-climate models (REMIND-MAgPIE) with additional models was applied to quantify sustainable development pathways using 56 SDG indicators and proxies under six policies (development, resource efficiency, climate change mitigation, food and energy, global equity, equality and poverty reduction) and multiple scenarios.^11^ The results show that additional actions (international climate finance, redistribution of carbon pricing revenues, healthy diets, and others) are needed to achieve climate and SDG goals and that some SDG gaps can be closed by 2050. The Global Change Analysis Model (GCAM) was applied to analyze regional and global SDG interactions arising from decarbonization pathways in pursuit of the Paris Agreement and to identify mitigation solutions.^10^ Complex IAMs are applied for *ex ante* assessments to project GHG emission reduction pathways and economic costs of climate policies in the European Union for 2030 and 2050 scenarios, using seven models with a wide range of SDG outcomes: partial equilibrium model (GCAM), two computable general equilibrium models (GEMINI-E3, ICES-XPS), a macro-econometric model (NEMESIS), an energy system model (EU-TIMES) and two sectoral models covering buildings, industry (FORECAST) and transport (ALADIN) in Europe; GCAM is also coupled with an atmospheric model for air quality and short-lived climate pollutants (TM5-FASST) and a global climate-carbon cycle model (Hector).^21^ The results provide insights into the co-benefits and side effects of mitigation and decarbonization policies on SDG 8 (economy) and SDG 2 (food prices, poverty, malnutrition), as well as potential policies to redistribute carbon tax revenues to compensate low-income households (ibid).

The main challenges of IAMs are model validation, analysis of causal pathways in large systems,^24^ data availability and data sources,^52^ oversimplification in proxy selection due to data availability and whether these proxies measure what they are intended to measure,^11^ scenario assumptions (or lack of explanation of scenarios and their assumptions), and general conclusions with high uncertainties due to model capabilities.^10^ The quantification of institutions and good governance remains a challenge to include in IAMs and to consider as endogenous factors interventions.^11^ Yet, they are key to progress on many SDGs.^6^ Research applying IAMs is still evolving, with ongoing improvements in target selection.^6^^,^^11^^,^^24^ The findings from IAMs indicate that the 2030 deadline is insufficient for achieving the SDGs, highlighting the need for an extension, with the possibility of reaching the main SDGs by 2050.

## Discussion and prospects

Scholarly publications on SDG interactions have grown since 2015, but we are still in the logical reasoning stage rather than evidence-based interlinkages and addressing these trade-offs. The 2030 Agenda’s high dimensionality presents a significant challenge to research efforts, especially due to data availability and analysis. One of the challenges in assessing the interlinkages between SDGs is the context-specific nature of these interlinkages^4^^,^^27^^,^^57^; yet most studies have been conducted globally or regionally.^13^ Semantic and correlation analyses were widely used in the early stages; recent trends include applying mixed methods, network analysis, and IAMs.^13^^,^^14^^,^^26^ Future studies may benefit from employing various methods reviewed in this paper, depending on research objectives, disciplinary perspectives, data availability, and the level of analysis, considering the gaps, biases, and limitations mentioned previously. For instance, addressing the challenge of policy coherence and context-specificity requires considering stakeholders’ perspectives. However, expert-based assessments are time-consuming, subjective, and difficult to generalize for broader areas and compare with other cases. Exploring global trends in certain SDG interactions requires quantitative analysis and data availability. However, correlation analysis does not imply causality nor present the analysis’s directionality and strengths. We acknowledge that this review paper focuses on research methods rather than reviewing all papers about WEF nexus. We have used WEF nexus as an example in our paper. There are other methods available that are not discussed here, such as system modeling, quantitative modeling—such as input-output, system dynamics, and agent-based modeling—and multi-criteria analysis are also employed.^13^^,^^26^ While these methods are gaining popularity, they can be challenging due to issues with data availability, uncertainties, and the complexities of modeling.

Multidisciplinary and cross-disciplinary approaches and methods are increasingly used to assess SDG interactions. While these approaches provide a complex perspective for sustainable development, there is a risk of “paralysis by analysis” if research objectives are not clearly stated and the results and policy implications are not communicated effectively.^26^^,^^67^ Most studies remain exploratory objectives rather than explanatory. Questions often arise regarding research objectives when addressing societal challenges and the intended audience of these publications, whether they are other researchers or policymakers. If the latter, policy relevance, priorities, and implications for policymaking should be addressed. When articles mention policy challenges, they focus on policy integration and coherence, contextualizing SDG interactions from an integrated perspective. However, they do not address policy evaluation, monitoring, or innovation.^26^ Furthermore, when the analysis fails to consider spatial and temporal scales, the description of SDG interactions becomes vague and abstract.

In analyzing SDG interlinkages, the literature^3^^,^^5^ shows that synergies outnumber trade-offs in sheer quantity, that is, nS > nT. Where nS denotes the number of synergy-related links and nT the number of trade-off-related links. However, there is a noteworthy gap in addressing the potential adverse impacts stemming from a greater cumulative strength in trade-offs compared to synergies. This is quantified by aggregating all weights (−3, −2 or −1) of trade-off related links (sT) and contrasting them with the sum of all weights (1, 2, or 3) from synergy-related links (sS). Consequently, the overall impact of having more instances of synergies than trade-offs may be counterbalanced if sS < sT. The current lack of trade-off studies may indicate low incentives and biases for reporting “negative” findings in research, their long-term nature, or a focus on the conceptualizing phase, and this issue should be explored further.^4^^,^^6^^,^^27^ Future SDG interaction studies should continue exploring interlinkages across various contexts and domains with clear statement of the research objectives, the level of analysis (both geographical and temporal), policy problems, and implications. Research methods should be developed to reveal the directionality of SDG interactions, the strength of interaction, and uncertainties in their analysis.

The current literature on SDG interactions is based solely on historical data and limited to consider future interlinkage projections, such as transboundary and intergenerational spillovers.^2^^,^^13^^,^^39^^,^^68^ Transboundary spillovers refer to the positive or negative effects of the progress of a certain country toward the 2030 Agenda, which may impact another country’s progress. Quantification of transboundary SDG interactions for 121 countries among 768 pairs of SDG indicators suggests synergetic linkages of human-caused flows (e.g., international trade) and nature-caused flows (e.g., river flow, air flow).^39^ However, the analysis is based on historical data, and limited or missing data on environmental degradation and the complexity of transboundary SDG interactions should be further explored. As Bennich et al.^26^ argue, there is a need for further exploration of the role of environmental SDGs in the overall progress of the 2030 Agenda. Also, the study reports that transboundary trade-offs are higher among neighboring countries than non-neighboring, which requires strengthening regional cooperation to address transboundary synergies and trade-offs.^39^ Consequently, limited or lack of actions to address water, energy, land, air pollution might have not only trade-offs on a national level but also on transboundary levels, and not only for the current generation but also for future generations. Intergenerational spillovers refer to the impact of past and current generations on future generations, such as climate change, water pollution, environmental degradation, and biodiversity loss among others. These issues may not be immediately visible, but they will cause significant costs for future generations. For example, Rockström et al.^68^ suggest protecting the global water cycle for all people and generations and acknowledging the impact of (in)actions on one place to another, especially landlocked countries. In the WEF nexus, a short-term synergy could be exemplified by constructing a hydropower dam, initially providing renewable energy and water for irrigation, thus improving agricultural productivity. However, long-term trade-offs exist as the dam disrupts water flow downstream, affecting the dynamics of ecosystems such as freshwater fisheries. With the effects of climate change, the construction of large dams may not be cost-effective. Hence, a global governance to address the intergenerational equity and planetary boundaries should be explored further.^2^

As mentioned in the World Conservation Strategy,^69^ in the reports of the World Commission on Environment and Development^70^ and the United Nations Conference on Environment and Development in Rio de Janeiro,^71^ earlier definitions of sustainable development emphasized intergenerational aspects and the needs of future generations. This was later framed as a holistic approach if goals and targets mutually support and lead toward sustainable development, which should be reconsidered but keeping the universality principle of the 2030 Agenda. The necessity for more holistic approaches, coupled with simultaneous multiple crises, requires transformation of certain SDGs and targets, including better integration of climate action, strong coordination and policy coherence, and applicability of the target space on local context.^2^^,^^6^^,^^10^^,^^11^^,^^21^^,^^72^ Selection of targets and indicators should have societal relevance, science based, valid for 2030 and beyond, quantifiable, transparent, actionable, achievable, and with availability of data and knowledge.^2^^,^^6^ These initiatives could foster a solid framework for the conceptualization and operationalization of SDG interlinkages, thereby enhancing results’ scalability, replicability, and comparability.

## Acknowledgments

A.A. and J.M. acknowledge the funding by the German Academic Exchange Service (DAAD) from funds of the Federal Ministry for Economic Cooperation (BMZ), SDG^nexus^ Network (no. 57526248), program “exceed—Hochschulexzellenz in der Entwicklungszusammenarbeit.”

## Author contributions

A.A. and J.M.: conceptualization, writing original draft, review, editing.

## Declaration of interests

The authors declare no competing interests.
