# Supplementary material for: Methodological approaches on synergies and trade-offs within the 2030 Agenda
Source: iScience. 2024 Oct 5;27(11):111100. doi: 10.1016/j.isci.2024.111100 (PMC11563936; doi:10.1016/j.isci.2024.111100)
Supplement: Document S2. Appendix B [file mmc2.pdf]

Appendix B. List of some databases for data, monitoring, and analysis on the SDGs

Databases	Description
SDG Global Database	This database provides data on the global indicators used to monitor progress towards sustainable development.
Sustainable Development Report	This report provides data for the annual assessment of progress made by all UN member states towards the Sustainable Development Goals, along with insights into critical areas requiring urgent action to accelerate progress.
Sustainable Development Report - Regional level	Examples of access to data for Sustainable Development Reports covering Latin American and Asian countries.
DataBank	The World Development Indicators database offers a wide range of data on development, including many indicators relevant to the SDGs, such as poverty, education, health, and environmental sustainability.
OECD Statistics	This database offers internationally comparable statistics on various social, economic, and environmental topics relevant to sustainable development.
The Global Health Observatory	This database is a comprehensive source of information and analyses on global health-related indicators relevant to several SDGs.
UNICEF Data	This database provides data on global child-related indicators, which is crucial for monitoring progress towards several SDGs.
Land Portal - SDG Land Tracker	This database provides easy access to official data and information on all land-specific SDG indicators. It concisely explains the indicators and why they are important, and tracks progress.
FAOSTAT	This database provides free access to food and agriculture data for over 245 countries and territories and covers all FAO regional groupings from 1961 to the most recent year available.
Global Biodiversity Information Facility	This database provides access to biodiversity data essential for understanding biodiversity patterns and trends, supporting conservation efforts, and monitoring progress towards sustainable development.
StaTact	It is a tool that provides cost-effective solutions for resolving data gaps through better-governed data ecosystems and improved data processes to monitor Sustainable Development Goals.
**Network analysis**	
SDG Interlinkages Analysis and Visualisation Tool	Quantitative SDG interlinkages at the national level for 27 countries in Asia and Africa.
Igraph	In this R package, one can find an open-source library for the analysis of networks. It includes many centrality metrics such as eigenvector, betweenness, degree, etc.
Signnet	One can find an open-source library for analyzing signed networks in this R package. It includes centrality metrics such as PN centrality.
pcalg, iota, sparsebn, CCM	These R packages provide all the tools required to estimate networks based on development-indicator data, causal inference, Bayesian networks, correlation classification methods, etc.
**Quantitative methods**	
SDG keywords mapping tool	SDG Keywords Dictionary Project seeks to build on the processes developed by the United Nations to create an expanded list of keywords that can be used to identify SDG-relevant research.
SDG mapper	This tool analyses the alignment of selected documents with the 2030 Agenda and its SDGs by uploading them into the application.
CMUSustainability/ SDGmapR	In this R package, one can find publicly available SDG keyword datasets and several functions related to text mapping to particular sets of keywords.
