# Supplementary material for: Methodological approaches on synergies and trade-offs within the 2030 Agenda
Source: iScience. 2024 Oct 5;27(11):111100. doi: 10.1016/j.isci.2024.111100 (PMC11563936; doi:10.1016/j.isci.2024.111100)
Supplement: Document S1. Appendix A [file mmc1.pdf]

iScience, Volume *27*

Supplemental information

Methodological approaches on synergies and trade-offs within the 2030 Agenda

Aliya Assubayeva and Jorge Marco

Appendix A. Seven-point scale for evaluating interactions among pairs of SDGs/targets. Adapted from Nilsson et al. (2016)[55]

Strength	Name	Interpretation	Criteria
**3**	**Indivisible**	High-level synergy intertwines the performance of one target with another, resulting in a superior overall performance that exceeds what could be achieved independently	Quantitative methods:<br>– Regressions demonstrate significance, with the contribution of the coefficient to the variable of interest being notably high.<br>– Correlation (Pearson or Spearman) coefficient falls within the range of 0.8 to 1, indicating a strong association.<br>– Comparative statistics - the observed percentage change in the variable of interest resulting from a shift in the affecting variable is notably high or very high.<br>Qualitative methods:<br>– Literature review - a substantial body of research has extensively investigated the interaction, consistently providing robust evidence supporting its strength.<br>– Experts' assessments are based on expertise, perceptions, experience, background, and observations.
**2**	**Reinforcing**	Moderate-level synergy creates conditions to improve the performance of the other target, leading to a more effective outcome than individual efforts	Quantitative methods:<br>– Regressions demonstrate that the influence of the coefficient on the variable of interest is of moderate significance.<br>– Correlation coefficient falls within the range of 0.6 to 0.8, indicating a moderate association.<br>– Comparative statistics - the observed percentage change in the variable of interest resulting from a shift in the affecting variable is of moderate magnitude.<br>Qualitative methods:<br>– Literature review - a body of research has partially explored the interaction, offering moderate evidence to support its strength.<br>– Experts' assessments are based on expertise, perceptions, experience, background, and observations.
**1**	**Enabling**	Low-level synergy results in marginal or minimal improvement over the other target, potentially lacking significant integration in the interaction	Quantitative methods:<br>– Regressions demonstrate that the influence of the coefficient on the variable of interest is low.<br>– Correlation coefficient falls within the range of 0.3 to 0.6, suggesting a weak association.<br>– Comparative statistics - the observed percentage change in the variable of interest resulting from a shift in the affecting variable is marginal or minimal.<br>Qualitative methods:<br>– Literature review - the interaction has been inadequately explored in the existing body of research, providing limited evidence to support its strength.<br>– Experts' assessments are based on expertise, perceptions, experience, background, and observations.

**0**	**Consistent**	No evidence of synergy or trade-off exists. Either no interactions are documented, or the interactions are ambiguously identified in the existing literature	Quantitative methods:<br>– Regressions demonstrate that the coefficient exerts no influence on the variable of interest.<br>– Correlation coefficient falls within the range of 0 to 0.3, indicating a lack of significance in the interaction.<br>– Comparative statistics - there is no observed percentage of change, or its determination is ambiguous and contingent on parameter values.<br>Qualitative methods:<br>– Literature review - the interaction remains unexplored in the existing literature or ambiguous results.<br>– Experts' assessments are based on expertise, perceptions, experience, background, and observations.
**-1**	**Constraining**	A low-level trade-off implies a minimal or marginal compromise between conflicting targets, leading to a limited decline in overall performance	Quantitative methods:<br>– Regressions demonstrate that the influence of the coefficient on the variable of interest is low.<br>Correlation coefficient falls within the range of -0.3 to -0.6, suggesting a weak association.<br>– Comparative statistics - the observed percentage of change is marginal or minimal.<br>Qualitative methods:<br>– Literature review - the interaction has been inadequately explored in the existing body of research, providing limited evidence to support its strength.<br>– Experts' assessments are based on expertise, perceptions, experience, background, and observations.
**-2**	**Counteracting**	Moderate-level trade-off involves a discernible compromise between conflicting targets, resulting in a moderate decline in overall performance	Quantitative methods:<br>– Regressions demonstrate that the influence of the coefficient on the variable of interest is of moderate significance.<br>– Correlation coefficient falls within the range of -0.6 to -0.8, indicating a moderate association.<br>– Comparative statistics - the observed percentage of change is of a moderate magnitude.<br>Qualitative methods:<br>– Literature review - research has partially explored the interaction, offering moderate evidence to support its strength.<br>– Experts' assessments are based on expertise, perceptions, experience, background, and observations.
**-3**	**Cancelling**	A high-level trade-off represents a substantial compromise between conflicting targets. Enhancing the performance of one target severely undermines the achievement of the other	Quantitative methods:<br>– Regressions demonstrate that the contribution of the coefficient to the variable of interest is notably high.<br>– Correlation coefficient falls within the range of -0.8 to -1, indicating a strong association.<br>– Comparative statistics - the observed percentage of change is notably high or very high.<br>Qualitative methods:<br>– Literature review - research has extensively investigated the negative interaction, consistently providing robust evidence supporting its strength.<br>– Experts' assessments are based on expertise, perceptions, experience, background, and observations.
